# From an Insect’s Point of View

**Published:** 1881-06

**Authors:** 


### FROM AN INSECT'S POINT OF VIEW.

WHAT a horrible place must this world appear when regarded according to our ideas from an insect's point of view! The air infested with huge flying hungry dragons, whose gaping and snapping mouths are ever intent upon swallowing the innocent creatures for whom, according to the insect, if he were like us, a properly constructed world ought to be exclusively adapted. The solid earth continually shaken by the approaching tread of hideous giants—moving mountains—that crush out precious lives at every footstep, an occasional draught of the blood of these monsters, stolen at life-risk, affording but poor compensation for such fatal persecution.

Let us hope that the little victims are less like ourselves than the doings of ants and bees might lead us to suppose; that their mental anxieties are not proportioned to the optical vigilance indicated by the four thousand eye-lenses of the common house-fly, the seventeen thousand of the cabbage butterfly and the wide-awake dragon-fly, or the twenty-five thousand possessed by certain species of still more vigilant beetles. The insect must see a whole world of wonders of which we know little or nothing. True, we have microscopes, with which we can see one thing at a time if carefully laid upon the stage; but what is the finest instrument that Ross can produce compared to that with 25,000 object glasses, all of them probably achromatic, and each one a living instrument with its own nerve branch supplying a separate sensation? To creatures thus endowed with microscopic vision, a cloud of sandy dust must appear like an avalanche of massive rock fragments, and everything else proportionally monstrous.

Insects are probably acquainted with a whole world of physical facts of which we are utterly ignorant. Our auditory apparatus supplies us with a knowledge of sounds. What are these sounds? They are vibrations of matter which are capable of producing corresponding or sympathetic vibrations of the drums of our ears or the bones of our skull. When we carefully examine the subject, and count the number of vibrations that produce our world of sounds of varying pitch, we find that the human ear can only respond to a limited range of such vibrations. If they exceed three thousand per second, the sound becomes too shrill for average people to hear it, though some exceptional ears can take up pulsations or waves that succeed each other more rapidly than this.

Reasoning from the analogy of stretched strings and membranes, and of air vibrating in tubes, etc., we are justified in concluding that the smaller the drum or tube, the higher will be the note it produces when agitated, and the smaller and the more rapid the aerial wave to which it will respond. The drums of insect ears, and the tubes, etc., connected with them, are so minute that their world of sounds probably begins where ours ceases; that what appears to us as a continuous sound is to them a series of separated blows, just as vibrations of ten or twelve per second appear separated to us. We begin to hear such vibrations as continuous sounds when they amount to about thirty per second. The insect's continuous sound, probably, begins beyond three thousand. The blue-bottle may thus enjoy a whole world of exquisite music of which we know nothing.—*Belgravia.*

